# The Potential Influence of Residual or Recurrent Disease on Bevacizumab Treatment Efficacy in Ovarian Cancer: Current Evidence and Future Perspectives

**DOI:** 10.3390/cancers16051063

**Published:** 2024-03-05

**Authors:** Klaudia Żak, Małgorzata Satora, Ilona Skrabalak, Rafał Tarkowski, Marta Ostrowska-Leśko, Marcin Bobiński

**Affiliations:** 1 Department of Medical Chemistry, Medical University of Lublin, 20-059 Lublin, Poland; zakklaudia3@gmail.com; 2 I Chair and Department of Oncological Gynaecology and Gynaecology, Student Scientific Association, Medical University of Lublin, 20-059 Lublin, Poland; msatoraa@gmail.com; 3 I Chair and Department of Oncological Gynaecology and Gynaecology, Medical University of Lublin, 20-059 Lublin, Poland; ilona.skrabalak@umlub.pl (I.S.); rafal.tarkowski@umlub.pl (R.T.); 4 Chair and Department of Toxicology, Medical University of Lublin, 20-090 Lublin, Poland; marta.ostrowska-lesko@umlub.pl

**Keywords:** bevacizumab, ovarian cancer, angiogenesis, VEGF

## Abstract

**Simple Summary:**

Bevacizumab was approved by the European Medicines Agency in December 2011 and by the Food and Drug Administration in June 2018 as a treatment for patients with epithelial ovarian cancer. However, numerous questions persist regarding the optimal conditions under which bevacizumab exerts its most beneficial effects. One particular area of uncertainty concerns the presence and location of residual or recurrent disease, particularly within the context of lymph nodes, lymph vessels, and the process of lymphangiogenesis. The mechanism of action of bevacizumab involves the inhibition of blood vessel formation by binding to all vascular endothelial growth factor type A isoforms. However, it is important to note that lymphangiogenesis relies on other members of the vascular endothelial growth factor family—specifically types C and D—meaning that the creation of lymph vessels is not directly affected by bevacizumab. This distinction may have implications not only for the efficacy of bevacizumab treatment but also for the patterns of recurrences following such treatment.

**Abstract:**

There were high hopes for the new antiangiogenic medicament, bevacizumab, which could inhibit the creation of new blood vessels through binding to isoform A of vascular endothelial growth factor (VEGF). However, it is not only blood vessels that are responsible for tumor cell spread. During the process of tumor growth, lymphangiogenesis is mediated by other members of the VEGF family, specifically VEGF-C and VEGF-D, which act independent to bevacizumab. Therefore, based on the mechanism of bevacizumab action and the processes of angio- and lymphangiogenesis, we formed three hypotheses: (1) if the lymph nodes in primary ovarian cancers are metastatic, the outcome of bevacizumab treatment is worsened; (2) concerning the second-line treatment, bevacizumab will act in a weakened manner if recurrence occurs in lymph nodes as opposed to a local recurrence; (3) patients treated by bevacizumab are more likely to have recurrences in lymph nodes. These hypotheses raise the issue of the existing knowledge gap, which concerns the effect of bevacizumab on metastatic lymph nodes.

## 1. Introduction

Already in 1939, Ide et al. suggested that tumors may release specific factors with the aim to stimulate the growth of blood vessels [1]. After 32 years, Dr. Folkmann, a renowned scientist known as “the father of angiogenesis”, proposed in the esteemed “New England Journal of Medicine” that cancer cells may be effectively treated by blocking their blood supply, depriving them of essential nutrients. This idea of anti-angiogenesis as a cancer treatment approach focuses on inhibiting the entry of new blood vessels into the early tumor implantation. Dr Folkmann already knew that without new blood vessels, tumors cannot grow from a microscopic size of 1–2 mm^3^ [2]. However, was he aware that this would become possible and used in clinical practice within three decades?

Bevacizumab’s development required the diligent efforts of numerous researchers. In 1983, Senger et al. identified the factor present in tumor ascites fluid from guinea pigs, hamsters, and mice increasing microvascular permeability [3]. In 1989, Ferrara and Hanzel observed that pituitary follicular cells secrete a growth factor specific for vascular endothelial cells. They proposed to name it vascular endothelial growth factor (VEGF), which remains to this day [4]. In 1993, Kim et al. published a study in the scientific journal “Nature” reporting that the treatment with a monoclonal antibody specific for VEGF inhibited the growth of the tumors in laboratory conditions. Moreover, it was observed that the density of the vessels in the treated group was lower [5]. Therefore, subsequent research was more and more advanced until the first registration of the new drug by the FDA in 2004 [6].

An antiangiogenic medicine, bevacizumab, is the treatment option which changed and is still changing today’s oncology. Bevacizumab is an antibody which is used in more and more indications. In 2011, it was approved by the European Medicines Agency (EMA) and in 2018 [7] by the FDA as a treatment option for patients with stage III or IV epithelial ovarian cancer [8]. In this indication, it still raises a lot of controversies. One of these aspects concerns the impact of the residual disease location of ovarian cancer on bevacizumab treatment. Bevacizumab acts by specifically binding VEGF-A, which is the isoform that plays a crucial role in forming new blood vessels [7,9,10]. Meanwhile, in tumors located in lymphatic organs such as lymph nodes, nutrients are also delivered by lymphatic vessels. The formation of those vessels is regulated by the other members from the VEGF family, namely VEGF-C and VEGF-D. It is worth noting that these isoforms are not targeted by bevacizumab. This is what limits the efficacy of bevacizumab treatment. However, is it truly of significant importance?

## 2. Materials and Methods

The studies cited in the presented review were selected from the PUBMED, Google Scholar and Science Direct databases. Terms used by us were created by combining all words connected with bevacizumab and different types of gynecological cancers, which we describe in our manuscript, by using Boolean operator “OR”. Among these terms were for example “bevacizumab”, “bev”, and “antiangiogenic treatment”. Thereafter, we were added new words of interest with the use of the Boolean operator “AND”. These words were changed depending on the part of the manuscript we were working on. Among them were for example “ovarian cancer” and “endometrial cancer”. Moreover, we also used more specific terms relating to the mechanism of action of bevacizumab including “residual disease” and “lymph nodes”, which are connected to selected sections. Even if the paper was designed as a narrative review, we applied the rules of paper selection listed below.

Inclusion criteria:

- The types of included studies: clinical trials, retrospective studies, reviews, and metanalyses;
- More than one patient described in the study;
- No limitations for the year of publication were used.

Exclusion criteria:

- Articles not written in English,
- Conference abstracts only,
- Study cases, and
- Duplicated papers.

## 3. Process of Angiogenesis

In order to grow and progress, solid tumors must constantly receive nutrients and oxygen. Angiogenesis, which refers to forming new blood vessels from pre-existing ones, is essential for the development of solid tumors [9,11]. Physiologically, angiogenesis is an important process during embryogenesis. In an adult, in turn, it is a process that is usually active in wound healing [10]. Moreover, enhanced angiogenesis has been demonstrated in the process of cancerogenesis, as the formation of new blood vessels is crucial to the metabolic demands of the tumor [11]. Moreover, angiogenesis is involved in the development of various diseases and pathological conditions, including vascular inflammation, rheumatoid arthritis, psoriasis, gastritis, inflammatory bowel disease, ischemic heart disease, atherosclerosis, cyanotic heart defects, and diabetes [10,12,13].

The creation of new blood vessels is possible thanks to pro-angiogenic factors secreted by tumors, i.e., VEGFs. These molecules interact with endothelial cells and stimulate angiogenesis by binding to VEGF receptor tyrosine kinases (VEGFR1-3) [14,15]. Moreover, study results showed increased VEGF expression within solid tumors associated with a poor prognosis—metastatic breast cancer [16], renal cell carcinoma [17], metastatic colorectal cancer [18], ovarian cancer, and various other types [19]. In the context of angiogenesis two terms: vascular normalization and normalization widow the seems to be important. “Vascular normalization” is a phenomenon involving the morphological organization of vessels in the tumor and, therefore, a decrease in the pressure of the interstitial fluid. This hypothesis assumes the restoration of the balance between pro- and antiangiogenic factors after the use of antiangiogenic therapy, targeting the metabolism of endothelial cells, which results in the restoration of the proper structure and function of vessels. Therefore, it is nothing more than restoring the incorrect functioning of cancer vessels in the tumor. This concerns the maturity of the vessels, more uniform blood flow and reduced vascular permeability or hypoxia [20]. The “normalization window” is a transient state that disappears with the action of the antiangiogenic drug. This is a time window during which tumor vessels have a less dense and more linear texture [21,22]. Taking into account that the overexpression of VEGF in cancers or metastases, promotes the formation of new blood vessels and, hence, tumor development, it seemed necessary to develop therapeutic methods targeting the antiangiogenic mechanism.

**Figure 1:**
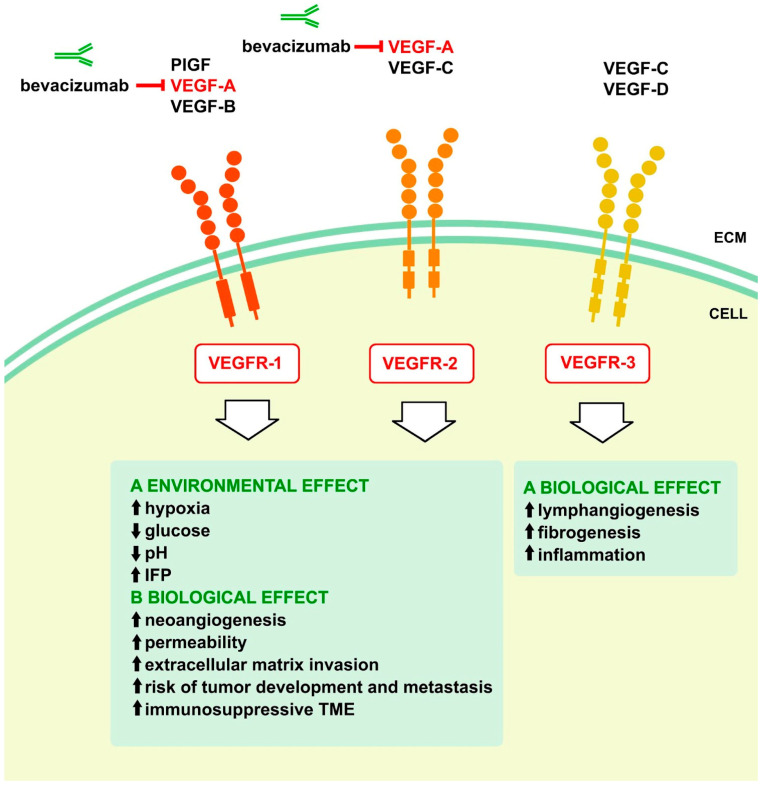
Vascular endothelial growth factor (VEGF) family members impact on tumor microenvironment. (ECM—extracellular matrix, IFP—interstitial fluid pressure, PIGF—placenta growth factor, TME—tumor microenvironment, and VEGFR—vascular endothelial growth factor receptor.)

Solid tumors consist of neoplastic cells and the surrounding stromal cells, such as tumor-associated fibroblasts, endothelial cells, pericytes, and vascular smooth muscle cells, making them intricate structures [23,24,25]. These cells play a crucial role in the growth and spread of tumors and their susceptibility to chemotherapy [23,24,26,27]. Furthermore, immune system cells, such as tumor-associated macrophages, are crucial in creating the inflammatory milieu within tumors. Cancer and stromal cells can release excessive proangiogenic substances, including VEGF, which in turn promotes environmental, vascular biological, and immunological effects [26,28,29] (Figure 1).

Due to reduced blood flow, tumors frequently experience hypoxia, leading to the continuous excessive synthesis of VEGF and persistent development of aberrant tumor blood vessel patterns [30]. The distribution of tumor blood vessels is dispersed, lacking a well-organized pattern of arterioles, capillaries, and venules. These vessels are characterized by variable and often enlarged diameters, irregular forms, leakage, and disordered blood flow [30]. The blood arteries in tumors are also poorly developed, with few loosely connected pericytes and smooth muscle cells, and lack complete basement membranes. The combination of these features and insufficient lymphatic drainage frequently results in impaired blood circulation and elevated interstitial fluid pressure (IFP) within the tumors [30]. Furthermore, VEGF triggers an immunosuppressive tumor microenvironment by impairing the maturation of dendritic cells, leading to the deactivation of cytotoxic T lymphocytes, promoting the production of regulatory T cells, tumor-associated macrophages, and myeloid-derived suppressor cells. In addition, VEGF increases the level of Programmed Death-1 in cytotoxic T lymphocytes and regulatory T cells [29,31].

## 4. Process of Lymphangiogenesis

**Figure 2:**
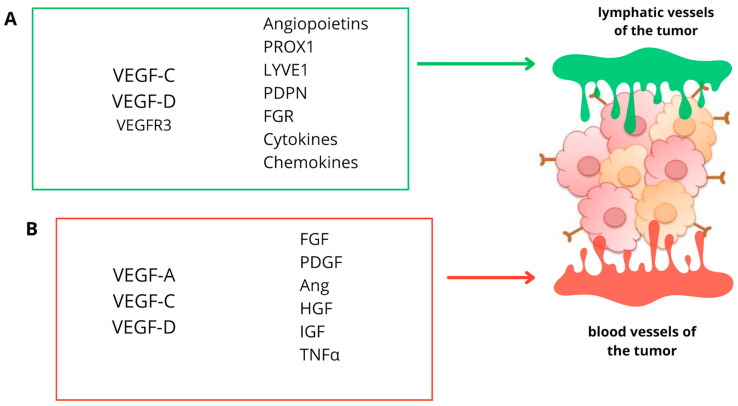
Factors involved in (**A**) lymphangiogenesis and (**B**) angiogenesis. (**A**) In the process of lymphangiogenesis, VEGF-C and VEGF-D play a key role, in addition, other factors influence the process of lymphangiogenesis. (**B**) In the process of angiogenesis, VEGF is one of the most well-studied growth factors; however, other factors, such as FGF, PDGF, Ang, HGF, IGF, and TNFα also play an essential role. (VEGF-C—vascular endothelial growth factor C, VEGF-D—vascular endothelial growth factor D, FGF—fibroblast growth factor, PDGF-platelet-derived growth factor, Ang—angiopoietin 1, HGF—hepatocyte growth factor, IGF—insulin-like growth factor, and TNFα—tumor necrosis factor α).

The creation of new lymphatic vessels from an existing system is known as lymphangiogenesis. This process depends on the interaction of the vascular endothelium with signaling molecules originating from serum and extracellular matrix (ECM) [32]. In conditions such as inflammation, tumor growth, or tissue repair, pathogenic lymphatic vessels originate from pre-existing vessels [33]. Lymphatic vessels are also responsible for cancer spread. Furthermore, they actively drive tumor cell recruitment to lymph nodes (LN), cancer stem cell survival and immunological regulation. Malignant tumors can produce factors that stimulate lymphatic vessel growth. In the study by Sopo et al., the significance of blood and lymphatic vessels in the prognosis of ovarian cancer was analyzed. The authors observed that the small size of lymph vessels was connected with 26% shorter 5-year survival. Moreover, the more lymphatic vessels were in the tumor, the more lymph node metastasis were present [34]. It has been proven that lymphangiogenesis is primarily mediated by the growth factors VEGF-C and VEGF-D, as well as the particular receptor VEGFR-3. This phenomenon is also induced by the endothelial lymphatic vascular hyaluronate receptor 1 (LYVE1), the prospero homeobox-related transcription factor 1 (PROX1), and the membrane glycoprotein—podoplanin (PDPN). Surprisingly, the latter two components were discovered to be expressed only in lymphatic vessels but not in blood vessels, so that they may be more specific for the lymphatic system [35]. Proving that hypoxia-induced regulation of lymphangiogenic factors made it possible to consider tumor hypoxia and lymphangiogenesis as closely related phenomena [36]. Lymphangiogenesis is not only regulated by VEGF-C and VEGF-D, which act directly on lymphendothelial progenitor cells (LECs), but also by other cytokines that may act indirectly by promoting VEGF-C expression. These include, for example, angiopoietins, which act directly or familial fibroblast growth factor (FGR), pro-inflammatory cytokines such as IL-1β and hepatocyte growth factor, platelet-derived growth factors, insulin-like growth factor 1 and 2, adrenomedullin and endothelin-1. Therefore, VEGF-C stands out as a key regulator of lymphangiogenesis (Figure 2).

As seen in ovarian cancer patients, tumor-associated lymphangiogenesis may increase metastasis and, as a consequence, decrease survival. Lymphatic capillaries at the tumor site let tumor cells metastasize from tumor foci to draining lymph nodes and enter the systemic circulation via nodal lymphatic vessels or blood vessels. The newly generated lymphatic capillaries promote the tumor’s inflammatory milieu and signal lymphatic vessel formation in this multistep process. Tumor-associated fibroblasts at the primary tumor site can secrete lymphangiogenic factors like VEGF-C, COX-2, HIF1 alpha chemokines like CCL21, CXCL12, which affect lymphatic endothelial cell permeability, adhesion molecule expression, migration to form nascent capillaries, and ECM remodeling [37].

Despite the fact that there are more factors that contribute to lymphangiogenesis, the two most important are VEGF-C and VEGF-D. All of these factors act as lymphangiogenic growth factors [32]. Overexpression of VEGF-C or VEGF-D has been found in studies describing tumor lymphangiogenesis to stimulate the development of tumor-associated lymphatic vessels, increase LN metastasis, and lead to a poor prognosis. VEGF-C or VEGF-D expression in primary tumors has been linked to LN metastasis in malignancies of the ovary, breast, colon, rectum, prostate, esophagus, stomach, lung, cervix, and endometrial [35]. Sopo et al. conducted a study that compared the expression levels of VEGF-C and VEGF-D in primary and metastatic cancers. VEGF-C expression was shown to be much higher in primary tumors, while VEGF-D expression was found to be higher in metastatic tumors [34]. A similar conclusion comes from a study on endometrial cancer, which demonstrates that only VEGF-D expression increases with cancer stage [38]. In well-differentiated colon cancer, there was equal overexpression of VEGF-C and VEGF-D. A significant association was discovered between the expression of lymphatic vessel growth factors and higher vessel density in the stroma surrounding the tumor, as well as a better prognosis [39].

## 5. Bevacizumab—Role in a Treatment

In 2004, the US FDA approved bevacizumab, the first antiangiogenic drug for the treatment of metastatic colon cancer [6]. This new antiangiogenic medicine is a humanized monoclonal antibody that has the ability to bind to all VEGF-A isoforms. This mechanism of bevacizumab prevents the activation of VEGF signaling pathways that would promote neovascularization. This prevents the formation of new blood vessels, which leads to the maturation of the tumor’s vascularization, preventing its excessive growth. Moreover, the antiangiogenic effect of bevacizumab has a synergistic effect with chemotherapy with carboplatin [40,41]. Since the first study on bevacizumab was published, numerous subsequent studies have been conducted describing the effects of bevacizumab in combination with cytotoxic chemotherapy in the treatment of solid tumors [40,42], including colorectal, lung, breast, and kidney cancer [43,44,45]. Since then, the literature reports that over 3,500,000 patients with severe cancer have been treated with bevacizumab [9].

Regarding the side effects of bevacizumab, the most common ones observed among patients are hypertension, weakness, abdominal pain and diarrhea [7]. Due to the risk of hypertension in patients, constant monitoring of blood pressure is necessary during bevacizumab therapy. Some patients with kidney cancer have developed proteinuria during treatment with this drug [9]. Studies have also described the occurrence of thromboembolism and an increased risk of hemorrhage in patients. In patients with colon or cervical cancer, there is a risk of gastrointestinal perforation. Most side effects occurred at the beginning of the treatment cycle when bevacizumab was administered together with chemotherapy. However, effects such as hypertension and proteinuria have been observed with long-term use of this drug [46,47].

The results of studies using bevacizumab in combination with other drugs and the immunomodulatory effect of bevacizumab seem to be promising. A phase III study evaluated bevacizumab in combination with atezolizumab in patients with non-small-cell lung cancer. Stratified OS was 64% in the bevacizumab plus atezolizumab group and 48% in the atezolizumab plus chemotherapy group. Regarding side effects, most were grade 1/2 immunological in nature [48]. To our knowledge, this is the first such study to evaluate the combination of bevacizumab with a drug that inhibits PD-L1/PD-1. It turned out that the addition of bevacizumab to atezolizumab is probably associated with a more favorable effect of atezolizumab at the level of PD-L1/PD-1 blockade in those patients with metastases who have not been previously treated with chemotherapy. A phase III study of the combination of bevacizumab with interferon α-2a in patients with metastatic renal cell carcinoma also showed an improvement in PFS in patients treated with this combination method compared to patients receiving interferon α-2a alone (10.2 months vs. 5.4 months). It seems interesting that the doses of interferon alfa in patients were reduced in order to avoid toxic side effects. The study results showed an improvement in PFS in those patients who received interferon alfa at a reduced dose [49]. The study results indicate that immune modulation with bevacizumab acting at the level of VEGF inhibition may be important in solid tumors with increased VEGF expression or liver metastases. Moreover, it turned out that the use of bevacizumab in combination with interferon alfa may lead to a reduction in interferon doses, which may prevent long-term side effects associated with it.

In the context of bevacizumab treatment, the aspect of the interstitial fluid pressure (IFP) also seems to be very important. Antiangiogenic drugs can reduce tumor vascular permeability and IFP by causing vessel “normalization”. This results in more efficient perfusion. Rakesh et al. discovered that antiangiogenic therapy increases interstitial convection within the tumor while decreasing fluid convection outside of the tumor edge. This causes greater drug convection within the tumor, decreased growth factor convection, lymphatic metastasis, and peritumor edema, all of which are serious complications of various cancers [50]. In addition, The Wu team created a vascular tumor growth model to show that hypertensive IFP serves as a physical barrier, preventing agents from entering the vascular system. They discovered that tumors with higher lymphatic resistance increase drug concentration quickly and wash out faster, whereas tumors with lower resistance accumulate less agents [51].

So, it is reasonable to assume that by eliminating abnormal blood vessels, antiangiogenic treatment can enhance tumor perfusion. The combination of antiangiogenic therapy with other medications is partially based on this premise. Interstitial pressure is decreased as a result of antiangiogenic therapy’s effects on tumor bulk reduction and perfusion improvement. Lymphatic veins might collapse due to high interstitial pressure. Therefore, by enhancing lymphatic permeability, antiangiogenic treatment can enhance lymphatic function and further decrease interstitial pressure.

High microvascular density (MVD) in primary tumors is linked to lymph node metastases, poor clinical outcomes, and metastatic disease. However, Rofsted et al. claim that hypoxic tissue in tumors is associated with metastatic disease and impaired survival. Tumors with high interstitial fluid pressure (IFP) showed hypoxic fractions, high MVD, lymphatic density, and elevated expression of VEGF-A and VEGF-C [52].

Taking into account numerous studies on bevacizumab, its antiangiogenic effect, effectiveness in cancer treatment, high safety profile and potential side effects, this drug still seems to be crucial in the treatment of patients with solid tumors.

## 6. Bevacizumab in the Treatment of Gynecological Cancers

Each year, approximately 2 million women are diagnosed with gynecological and breast cancer all over the world [53]. Taking into account the high number of patients diagnosed with those types of cancer, new drugs are still investigated. Bevacizumab is one of the drugs which seems to be a really helpful treatment option. The studies describing this direct VEGF inhibitor are described below.

### 6.1. Ovarian Cancer

Bevacizumab was approved by the EMA in December 2011 and in June 2018 by the FDA as a treatment for patients with stage III or IV epithelial ovarian cancer [54]. Patients may undergo chemotherapy with carboplatin, paclitaxel, and bevacizumab, as well as surgical resection. Standard treatment of patients with ovarian cancer with bevacizumab lasts 15 months [55,56].

The two most important studies describing the use of bevacizumab in the treatment of ovarian cancer, which guaranteed the approval of the FDA, are GOG-218 and ICON7 [56,57]. GOG-218, a double-blind, placebo-controlled phase III study, enrolled 1873 women. Patients were randomly assigned to three treatments. In each group, patients received chemotherapy in the form of paclitaxel and carboplatin. The control group received chemotherapy plus a placebo in cycles 2 through 22. In the bevacizumab initiation group, patients received chemotherapy plus bevacizumab at a dose of 15 mg/kg b.w. in cycles 2 through 6 and placebo in cycles 7 through 22. In the third group, patients received bevacizumab in cycles 2 to 22. The median progression-free survival (PFS) in patients taking bevacizumab was 14.1 months in the group receiving bevacizumab continuously compared with PFS of 11.2 months in the group receiving bevacizumab at the beginning and 10.3 months in the control group [55].

ICON7 was an international, open-label, randomized phase III trial that demonstrated improved PFS when bevacizumab was added to chemotherapy in patients with high-risk early-stage or advanced-stage ovarian cancer. A total of 1528 women in the study were randomized to chemotherapy alone (*n* = 764) or chemotherapy plus bevacizumab 7.5 mg per kg intravenously every 3 weeks for 12 3-week maintenance cycles (*n* = 764). Overall survival was 45.4 months in the chemotherapy plus bevacizumab group and 44.6 months in the chemotherapy alone group. However, in the group of 502 patients with poor prognosis, the mean PFS was 34.5 months with standard chemotherapy and 36.3 months with chemotherapy in combination with bevacizumab. Although the use of bevacizumab did not increase the mean PFS in patients in the entire group, an increase in PFS was observed in patients with a poor prognosis. The study results emphasize a significant relationship between a higher stage of disease and a more beneficial effect of bevacizumab. There is a group of women who will not benefit from treatment with bevacizumab—these are mainly patients in FIGO stage I/II. Given the effects of bevacizumab on the tumor microenvironment, it appears that bevacizumab is effective in residual physical tumor that produces VEGF [57].

Interestingly, bevacizumab has shown synergy with other treatments in patients with ovarian cancer in many studies. Studies indicate that bevacizumab added to carboplatin in combination with paclitaxel contributed to the prolongation of PFS [56,57]. The combination of bevacizumab with PARP inhibitors is also interesting. In 2023, the final results of the randomized, double-blind, phase III trial PAOLA-1 were published. The aim of the study was to evaluate the addition of olaparib to bevacizumab in patients with newly diagnosed ovarian cancer. A total of 537 patients were assigned to olaparib (300 mg twice daily for up to 24 months) plus bevacizumab (15 mg/kg every 3 weeks for 15 months) and 269 patients were assigned to placebo plus bevacizumab. Median OS was 56.5 months in the olaparib plus bevacizumab group and 51.6 months in the placebo group. Moreover, benefits have been demonstrated in patients with BRCAm knockout or homologous recombination deficiency genes (HRD). Significant improvement in 5-year OS was demonstrated in patients with HRD-positive ovarian cancer (65.5%) compared to patients with HRD-negative ovarian cancer (48.4%) [58]. Taking into account the fact that the addition of olaparib to bevacizumab resulted in prolonged OS in patients, there is a need to study biomarkers in patients with ovarian cancer to determine an appropriate treatment regimen.

Another study was the open-label randomized phase III trial AGO-OVAR 17 BOOS/GINECO OV118/ENGOT Ov-15, the aim of which was to compare a standard of 15 dosages and extended duration of bevacizumab treatment with chemotherapy in 927 patients with newly diagnosed stage IIB-IV ovarian cancer. The patients first underwent cytoreductive surgery with 6 cycles of chemotherapy in the form of paclitaxel at a dose of 175 mg/m^2^ with carboplatin area under the curve 5 times every 3 weeks and bevacizumab at a dose of 15 mg/kg once every 3 weeks. Patients received bevacizumab for 15 or 30 months. The median PFS was 24.2 months in patients treated with the standard duration and 26.0 months in patients treated with the extended duration. Regarding OS, no difference was found between patient groups [59]. The study results showed that the extension of bevacizumab treatment in patients with ovarian cancer did not significantly affect PFS and OS.

In 2023, the results of a study assessing the effectiveness and safety of the combination of mirvetuximab soravtensine and bevacizumab in patients with platinum-resistant ovarian cancer were published. A total of 94 patients qualified into the group receiving mirvetuximab soravtensine at a dose of 6 mg/kg adjusted ideal body weight and bevacizumab at a dose of 15 mg/kg intravenously once every 3 weeks. It seems significant that 52% of patients had previously received ≥3 therapies, 59% had previously received bevacizumab, and 27% had previously received PARP therapy. The median PFS was 8.2 months and the median duration of response (DOR) was 9.7 months. Given the good results with this combination, treatment with bevacizumab and mirvetuximab soravtensine appears to be promising. These results were favorable regardless of the level of folate receptor alpha (FRα) expression or previous treatment with bevacizumab [60].

Although bevacizumab, as an antiangiogenic drug, has been described in many studies, its use in patients with ovarian cancer is still controversial. This is evidenced by the results of the GOG-218 study, in which a significant improvement in PFS was observed in the group of patients with stage IV cancer. In 2022, You et al. conducted an external validation study in the GOG-218 study using the KELIM scale to determine factors influencing bevacizumab treatment. To assess the benefits using the KELIM scale, it is important to measure at least three CA125 values during the first three cycles of chemotherapy. A total of 1662 patients with 3 or more upper levels of norm (ULN) CA125 marker values were examined using the scale. The study showed that PFS and OS were higher in high-risk patients with poorly chemosensitive disease [61]. All clinical trials with bevacizumab in ovarian cancer are presented in Table 1. When qualifying a patient for bevacizumab treatment, it is necessary to not only take into account the risk of disease progression and response to previous treatment, but also the value of markers that may be important.

### 6.2. Cervical Cancer

Thanks to constant cytological tests and the increase in vaccinations against the human papillomavirus (HPV), a decrease in the number of cervical cancer cases has been observed in recent years. However, despite available diagnostic methods, 500,000 women worldwide will be diagnosed with cervical cancer each year [62,63]. Moreover, radical surgery and radiotherapy, to which patients in the early stages of cervical cancer respond well, do not provide such good treatment results in women with metastases or recurrent disease [64]. Chronic HPV infection may lead to inactivation of cancer-suppressor genes, including p53 and pRb. This causes the accumulation of HIF-1 protein, which in turn induces an increase in VEGF expression by the tumor tissue. Therefore, bevacizumab, by inhibiting VEGF, has been used in the treatment of cervical cancer [65,66].

GOG 227C, the phase II trial from 2009, assessed the treatment of 46 patients with recurrent squamous cell cervical cancer with bevacizumab at a single dose of 15 mg/kg body weight. Every 3 weeks. The median overall survival was 7.3 months, and the median progression-free survival was 3.4 months. In the study, there was no control group, but the results were compared with other studies [67,68,69,70,71,72]. A total of 24% of patients treated with bevacizumab did not progress after 6 months on protocol, which was the highest score in all analyzed researches [73].

In a 2014 study, Tewari et al. (GOG 240 study) evaluated the effectiveness of combination therapy with platinum-free chemotherapy and bevacizumab in 452 patients with recurrent (325 patients), persistent (51 patients), or metastatic (76 patients) cervical cancer. Patients were randomly assigned to receive chemotherapy with bevacizumab (227 women) or without (225 women). Chemotherapy as cisplatin and paclitaxel, or topotecan and paclitaxel was administered in 1 day. Cycles were repeated every 21 days. The study results showed that treatment with chemotherapy together with bevacizumab was associated with increased overall survival of patients (17.0 months) compared to treatment with chemotherapy alone (13.3 months) [74].

GOG 240 is a randomized, controlled, open-label, phase III trial that assessed the effectiveness of bevacizumab in 452 patients with advanced cervical cancer. Patients were assigned in a 1:1:1:1 ratio to receive chemotherapy with cisplatin plus paclitaxel or topotecan plus paclitaxel with or without bevacizumab administered intravenously at a dose of 15 mg/kg on day 1 in 21-day cycles. The median OS was 16.8 months in the chemotherapy plus bevacizumab group and 13.3 months in the chemotherapy alone group. OS after progression was 8.4 months in patients in the group receiving chemotherapy plus bevacizumab and 7.1 months in the group receiving chemotherapy alone. The study results confirmed the effectiveness of bevacizumab in patients with advanced cervical cancer [75].

The results of available studies unanimously indicate the beneficial effects of bevacizumab in patients with cervical cancer. For the moment, bevacizumab has been approved as an antiangiogenic therapy in patients with advanced tumors. Given the low effectiveness of bevacizumab in early stages of cancer, it does not seem necessary to perform it in patients with benign tumors. Moreover, it may be beneficial to combine bevacizumab with other new drugs. The BEATcc trial is currently being conducted—a randomized, open-label, multicenter phase III clinical trial. Its aim is to determine whether adding atezolizumab—the immune checkpoint inhibitor—to bevacizumab will improve survival in patients with cervical cancer [76]. The results of KEYNOTE-826—a multicenter, randomized, phase III trial, seem to be also interesting, in which the authors assessed the effectiveness and improvement of quality of life after adding pembrolizumab to chemotherapy with or without bevacizumab. This study was conducted on 617 patients with persistent, recurrent or metastatic cervical cancer not previously treated with systemic chemotherapy. Patients were assigned to receive pembrolizumab 200 mg or placebo intravenously every 3 weeks and chemotherapy (paclitaxel or carboplatin) with or without bevacizumab 15 mg/kg every 3 weeks intravenously. GHS quality of life improved in 122 of 290 patients treated in the pembrolizumab group compared to 85 of 297 patients treated in the placebo group. The study results indicate an improvement in the quality of life after adding pembrolizumab to treatment, which indicates the benefits of treatment with immunotherapy [77]. All studies describing clinical trials with bevacizumab in the cervical cancer are presented in Table 2.

### 6.3. Endometrial Cancer

Endometrial cancer continues to be the most common cancer among women in the developed countries, and its incidence is increasing each year compared with other gynecological cancers, which are decreasing in number [78]. Chemotherapeutics, such as paclitaxel and carboplatin, are used as initial treatment for advanced endometrial cancer. When it comes to adjuvant therapy, it mainly includes surgical, hormonal and chemotherapy methods used depending on the histological grade and stage of the tumor [79]. Therefore, antiangiogenic drugs seemed to be an excellent option, but did it turn out to be true?

In 2007, Wright et al. retrospectively analyzed 11 patients with recurrent endometrial cancer treated with bevacizumab. Among the group of patients, nine women were patients with epithelial endometrial cancer, and two were patients with globular cell sarcoma. A total of three patients were in stage I, one patient in stage II, two patients in stage III and five patients in stage IV. All patients were previously treated with three chemotherapy regimens and received bevacizumab in combination with a chemotherapy agent. The median progression-free period for all patients was 5.4 months, including 8.7 months for five patients. Unfortunately, the study was performed on a small group of patients and there was no control group; therefore, it is difficult to compare the results [80].

Already in 2011, Aghajanian et al. conducted a phase II study to determine the tolerability of bevacizumab in the treatment of recurrent or persistent endometrial cancer. A total of 52 patients were treated with bevacizumab at a dose of 15 mg/kg administered intravenously every 3 weeks. In 21 patients (40.4%), PFS was at least 6 months. The median survival time was 4.2 months and the median overall survival time was 10.5 months. At that stage, it was recognized that due to prolonged PFS, this drug is worthy of further investigation in endometrial cancer [81].

Alvarez et al. conducted a phase II study, whose aim was to evaluate the combination of temsirolimus, an inhibitor of mTOR, and bevacizumab in patients with recurrent or persistent endometrial cancer. A total of 53 patients were enrolled in the study, of which 40 patients had previously received treatment with one chemotherapy regimen, 9 patients with two regimens, and 20 patients had been previously treated with radiotherapy. Patients received treatment with bevacizumab 10 mg/kg every other week and temsirolimus 25 mg intravenously weekly until disease progression. The median progression-free survival was 5.6 months and the median overall survival was 16.9 months. In this study, no control group was included. Moreover, toxicity seemed to be a great issue, because it was present in nearly 40% of patients [82].

Rubinstein et al., in a retrospective study, showed moderate effectiveness of bevacizumab in monotherapy in the treatment of advanced endometrial cancer. A total of 101 patients were included in the study, including 46% of cases in the stage IV according to FIGO and 24% in stage III. In addition, patients had differential histology, the most common of which was serous histology in 44%. Patients were assigned to appropriate cohorts based on prior treatment: cohort I included 14 patients who had previously received 1 line of treatment, cohort II included 29 patients who had previously received 2 lines of treatment, cohort III included patients who had previously received 3 lines of treatment, and cohort IV included patients who had received ≥4 lines of treatment. What is interesting, the group who received ≥4 lines of treatment had the highest median PFS: 4.9 months compared to cohort I: 3.5 months, cohort II: 2.6 months, cohort III 3.5 months. Therefore, the authors of the study opt that bevacizumab, due to an excellent result in the group after other lines of treatment, should be considered as a palliative treatment [83].

However, no randomized study reported prolonged PFS. In 2018, the first results of the GOG 209 trial were published—a randomized phase II trial. The study included 349 patients with chemotherapy-naïve stage III/IVA and IVB or recurrent endometrial cancer. Patients were randomly assigned to receive paclitaxel and carboplatin with bevacizumab, carboplatin with temsirolimus, or ixabepilone and carboplatin with bevacizumab. The study results did not show an increase in PFS in the group of patients treated with bevacizumab compared to the group of patients treated with carboplatin plus temsirolimus and the group of patients treated with ixabepilone and carboplatin. The hazard ratios for the groups were 0.81, 1.22 and 0.87, respectively [84].

For the moment, no significant profit of bevacizumab in the endometrial cancer treatment was observed, therefore this drug is not used in daily clinical practice. It is also probably connected with the fact that generally endometrial cancer is diagnosed in not advanced stages and surgery is the most effective treatment option, so the potential benefit from antiangiogenic treatment might be seen only in advanced/recurrent cases. A meta-analysis from 2020 showed that conventional chemotherapy in combination with bevacizumab improved median PFS and OS in patients with advanced or recurrent endometrial cancer [85]. All clinical trials with bevacizumab in the endometrial cancer are analyzed in Table 3.

## 7. Bevacizumab in Primary Ovarian Cancer Treatment and Maintenance and the Potential Impact of Residual Disease and Its Characteristics

Already in 2011, bevacizumab was approved by the EMA in the treatment of patients with epithelial ovarian, fallopian tube or primary peritoneal cancer in combination with carboplatin and paclitaxel for stage III or IV disease after initial surgical resection by the FDA [8]. As it was proven, this antiangiogenic drug did not increase overall survival (OS) in the whole population of cancer patients, but the difference in OS was observed in the poor-prognosis patients [57,86]. The definition of high-risk patients, meaning the patients with the worst prognosis, in ICON7 and GOG-128 studies have some differences. In the ICON7 study, the definition was as follows: stage IV or inoperable stage III disease, or suboptimally debulked (>1 cm) stage III ovarian cancer [57]. The GOG-218 study included patients in stage III with optimal (≤1 cm) and suboptimal (>1 cm) cytoreduction and stage IV [86]. However, in the aim to facilitate the comparison of both studies, some additional exploratory analyses were performed in the GOG-218 study, and as a consequence both studies had the same result: no OS benefit and PFS benefit [57,86]. The question is why bevacizumab brings the best results when it is used in patients with the worst prognosis? In general, the best prognosis for patients with ovarian cancer is ensured due to complete surgical resection. The fact that the optimal residual disease is the best alternative in the cases when complete surgical resection is not possible is also reported by many studies. But what does it mean that resection is optimal? The definition says that the largest disease diameter is less than 1 cm, independent of the total volume of disease [87,88,89,90,91,92,93,94]. The study of Manning-Geist et al. showed that the median progression-free survival for the following groups: with complete resection, with ≤1 cm residual disease in a single anatomic location, ≤1 cm residual disease in a multiple anatomic location and in the group suboptimally debulked were accordingly 14, 12, 10 and 6 months. The median overall survival in the above-mentioned groups were, respectively, 58, 37, 26 and 33 months [95]. Taking into account aforementioned information, in the cases of ovarian cancer the maximum surgical effort is required to achieve the lowest number of visible lesions. The minimal residual disease is clinically undetectable disease, a small number of cancer cells left in the body after treatment. Those cells are not connected with vessels, and this is one of the reasons why they are less sensitive to anti-VEGF therapy—no vessels, no drug action point and thus no effect of bevacizumab treatment. Especially compared to the lesions which are visible and connected by vessels—macroscopic disease [13,96,97]. This is the reason why bevacizumab may not change OS or PFS in the non-high-risk patients. However, in patients with no visible lesions, bevacizumab is used as angioprevention, which means stopping new blood vessel creation. In the study by Albini et al., bevacizumab is one of the treatments which should be considered in patients who have achieved cancer remission. In this regard, the main aim of medication is to prevent disease recurrences [98]. It is described as the suppression of the “soil”, meaning microenvironment, so that the “seed” (microscopic metastases) will not survive [99,100].

To the best of our knowledge, none of the studies analyzed the impact of the location of residual disease of ovarian cancer on bevacizumab treatment. According to the mechanism of bevacizumab [7,9,43,44,45,46,47,48,49,50,101], we know that this treatment acts by binding VEGF-A present in blood vessels, without binding VEGF-C and VEGF-D present in lymphatic vessels [30,31,32,33,34,35,36,37,38,39,102]. In this context, we would like to divide the presence of residual disease into nodal and not-nodal types. Potentially, bevacizumab should act well in the cases of not-nodal residual disease. Why? This is more probable that in the process of spreading the tumor, the new blood vessels will be formed. Blood vessels, which are more susceptible to the action of bevacizumab due to the presence of VEGF-A receptors. Compared to the nodal residual disease, in which the action of bevacizumab is limited, due to the presence of lymphangiogenesis regulated predominantly by VEGF-C and VEGF-D.

## 8. Bevacizumab in Recurrent Ovarian Cancer, Potential Impact of Recurrence Characteristics

There are three studies analyzing the efficacy of bevacizumab in the second-line treatment: OCEANS, AURELIA and GOG213. First of aforementioned, OCEANS analyzed the efficacy of bevacizumab in the dose of 15 mg/kg with gemcitabine and carboplatin in platinum-sensitive recurrent ovarian, primary peritoneal, or fallopian tube cancer. Median PFS was 12.4 in the bevacizumab-treated group vs. 8.4 months in the control group [103]. The study did not describe the location of the tumor recurrences, therefore we cannot state if this location has an impact on the bevacizumab treatment efficacy. The same conclusion relates to the AURELIA study, which analyzed the effectiveness of bevacizumab (10 mg/kg every 2 weeks or 15 mg/kg every 3 weeks) combined with chemotherapy in platinum-resistant ovarian cancer. Median PFS was almost two times longer in the bevacizumab group compared to the control group, 6.7 vs. 3.4 months, respectively [104]. GOG213 study analyzed the role of bevacizumab in the dosage of 15 mg/kg every 3 weeks in patients with recurrent platinum-sensitive ovarian cancer, who performed secondary surgical cytoreduction. The study showed that the median OS in the bevacizumab-treated group was 42.2 months and 37.3 in the chemotherapy alone group [105]. The question if it is better to use bevacizumab in the first- or in the second-line treatment was asked by many researchers, including Sznurkowski. In his review, he suggested that bevacizumab should be preserved for the second-line maintenance therapy, because it is more efficacious than in the first-line treatment [106]. It is consistent with the results of a meta-analysis, which implies that, compared to first-line treatment, in second-line treatment, bevacizumab shows significant survival benefit in PFS and OS in high-risk patients and patients with recurrences [107].

From our perspective, there is a knowledge gap in understanding why bevacizumab acts more successfully in patients with recurrences. At the beginning, we should recall the “traditional” recurrence patterns in ovarian cancer in the bevacizumab-non-treated group. Paik et al. conducted a study on the group of 303 patients with the epithelial ovarian cancer with no gross residual disease analyzed patterns of recurrence after primary debulking surgery. Firstly, the location of recurrence was analyzed. In the stages III–IV, distant recurrences were more frequent than locoregional recurrences—83.3% vs. 16.7%, accordingly. Secondly, discrete recurrences were more common than diffuse recurrences. The definition of diffuse carcinomatosis was as follows: nodules larger than four centimeters or constituting plaques on the peritoneum or mesentery. In stages III–IV, 39 patients (65%) had discrete recurrences and 21 (35%) had diffuse recurrences [108]. So, what are the consequences of bevacizumab use in the context of recurrences?

Taking into consideration the mechanism of bevacizumab, meaning inhibition of VEGF-A without affecting VEGF-C and VEGF-D, it seems to be clear that some differences in the patterns of recurrences, especially in the lymphatic system, may occur. The study by Petrillo et al. aimed to evaluate the timing and pattern of relapse in patients with advanced ovarian cancer treated in the first line by standard chemotherapy with or without bevacizumab. The research was performed on the group of 74 patients in the bevacizumab group and 148 patients in the chemotherapy alone group. What is interesting, even if the PFS was longer in the bevacizumab group, is that the recurrence pattern was different, especially in the context of lymph nodes. Patients treated with bevacizumab were more likely to relapse in lymph nodes—in the bevacizumab group metastases were present in 51.3% of patients compared to the control group: 31.1% of patients (*p* = 0.004). Moreover, peritoneal recurrence described as diffuse disease was also more frequent in patients treated in the first line by bevacizumab (96.8%) compared to the chemotherapy group [109]. The results of the study were consistent with previous study conducted by Dao et al. which showed, that patients treated with bevacizumab in the first line had a higher probability to recur in extra-visceral sites (*p* = 0.04) or in lymph nodes, especially those located extraperitoneally (*p* = 0.0002) [110]. In the comparison, the study by Kim et al. performed on the group of 52 patients with epithelial ovarian cancer treated by platinum-based doublet chemotherapy with bevacizumab as a second-line treatment and 104 patients in the control group only treated by chemotherapy did not show the same dependence. Bevacizumab-treated patients had a lower probability of developing disease recurrence in retroperitoneal lymph nodes (*p* = 0.001), pelvis (*p* = 0.003) and abdomen (*p* = 0.001), but the same for distant metastases (*p* = 0.32) compared to the control group. The authors of the study mentioned the heterogeneity in study design, study populations, and disease settings as the reason for the differences [111]. To the best of our knowledge, this is the only study in which the lower frequency of nodal metastases was observed.

## 9. The Potential Impact of Bevacizumab Treatment on Recurrence Patterns

The next question which appears during the analysis of the recurrence patterns after bevacizumab treatment is the following: how this process runs in other types of neoplasm. The systematic review and meta-analysis of 17 articles describing the recurrence patterns in high-grade glioma showed that in the bevacizumab-treated patients, the non-local recurrences were more frequent than in the standard-treated patients [112]. However, the results of the studies are not consistent, because the other two studies suggest that in glioma local progression after bevacizumab, treatment is more common [113,114]. Anyway the recurrence patterns in glioma, in our opinion, do not help us in understanding the ways of recurrence in ovarian cancer. How about other gynecological cancers? The analysis of the bevacizumab recurrence patterns in persistent recurrent or metastatic cervical cancer treated by bevacizumab in combination with cisplatin and paclitaxel showed that loco-regional recurrence was more frequent [115]. To the best of our knowledge, there are no studies describing the patterns of recurrence after bevacizumab treatment in endometrial cancer, which indicates the lack of knowledge in this area. From our point of view, the recurrence patterns after bevacizumab treatment in gliomas do not have an influence on the understanding of the recurrence patterns after bevacizumab treatment in ovarian cancer or other gynecologic neoplasms, because those types of cancer metastasize differently even without the antiangiogenic treatment.

## 10. Discussion and Conclusions

Bevacizumab, as an antiangiogenic drug, was created to interrupt angiogenesis by binding to VEGF-A isoform. As a consequence, a reduction in microvascular growth of tumor blood vessels is observed, which limits the blood supply to tumor tissues and ensuing tumor growth [7,9,40,41,43,44,45,46,101,116]. However, angiogenesis is not the only process which leads to tumor growth and metastases—equally important is lymphangiogenesis, which is also dependent on the VEGF family. According to Karaman et al., the process of lymphangiogenesis is mediated by other members of the VEGF family: VEGF-C and VEGF-D [35]. Those whose expression is elevated especially in lymph nodes are not affected by bevacizumab activity [33,34,35,36,37,38,39,40,41,42,102,117,118]. As a consequence, even if the creation of new blood vessels is interrupted by bevacizumab, the presence of lymphatic vessels is not reduced. Therefore, our first hypothesis is born: if the lymph nodes in primary ovarian cancers are metastatic, the outcome of bevacizumab treatment is worsened. The second one is connected with the first one, but it concerns second-line treatment. In our opinion, bevacizumab is going to act in a weakened manner if recurrence occurs in lymph nodes as opposed to a local recurrence. To this day, there are no studies analyzing the relationship between the presence of metastases in lymph nodes in primary ovarian cancer and the outcome of bevacizumab treatment. The bevacizumab approval in the treatment of patients with ovarian cancer refers to patients in stage III or IV disease after initial surgical resection. It is a very heterogeneous group, which may have both positive or negative lymph nodes. Moreover, there is a knowledge gap regarding the differences of bevacizumab efficacy in the second-line treatment depending on the location of recurrence locations. Our third hypothesis, which is partially confirmed, is the following: patients treated by bevacizumab are more likely to have recurrences in lymph nodes. Two out of three studies, performed by Petrillo et al. and Dao et al., showed that the patterns of recurrences are different when patients are treated by bevacizumab [109,110]. More precisely, in the Petrillo et al. study, metastases in lymph nodes were present in 51.3% of patients in the bevacizumab-treated group and 31.1% of patients in the control group [109]. Those conclusions were not in accordance with Kim et al., who observed that bevacizumab-treated patients had a lower probability of developing disease recurrence in retroperitoneal lymph nodes [111].

To conclude, the analysis of the current literature identifies a gap in knowledge about the potential influence of disease localization on the effect of bevacizumab treatment. This problem was not a part of wide clinical investigations so far and we can only base this on speculations. To answer the question if there are any differences in response to bevacizumab between nodal vs. no-nodal disease, this question must be included in future clinical trials.

## Figures and Tables

**Table 1:** Clinical trials with bevacizumab in ovarian cancer.

The Name of the Study	The Year of the Study	The Phase of the Study	Research Group	Dose of Bevacizumab	Results
GOG-218 [55]	2011	Phase III	1873 patients with ovarian cancer with newly diagnosed stage III (incompletely resectable) or stage IV epithelial ovarian cancer who had undergone debulking surgery to receive one of three treatments	Bevacizumab-initiation: chemotherapy + bevacizumab (15 mg/kg), cycles 2–6, placebo, cycles 7–22. Bevacizumab-throughout: chemotherapy + bevacizumab, cycles: 2–22.	Median PFS: control 10.3 months bevacizumab-initiation group: 11.2, bevacizumab-throughout group 14.1.
ICON-7 [57]	2015	Phase III	1528 patients with newly diagnosed ovarian cancer	Bevacizumab 7.5 mg/kg every 3 weeks, given concurrently and continued with up to 12 further 3-weekly cycles of maintenance therapy.	The mean PFS: chemotherapy + bevacizumab: 36.3 months, standard chemotherapy: 34.5 months. Median OS: chemotherapy + bevacizumab: 45.4 months, standard chemotherapy: 44.6 months.
PAOLA-1 [58]	2023	Phase III	809 patients with ovarian cancer	Olaparib (300 mg twice daily for up to 24 months) + bevacizumab (15 mg/kg every 3 weeks for 15 months); placebo group: bevacizumab alone	Median OS: olaparib + bevacizumab: 56.5 months, bevacizumab group: 51.6 months 5-year OS in patients with HRD-positive ovarian cancer (65.5%) compared to patients with HRD-negative ovarian cancer (48.4%)
AGO-OVAR 17 BOOS/GINECO OV118/ENGOT Ov-15 [59]	2023	Phase III	927 patients with newly diagnosed stage IIB–IV ovarian cancer	Bevacizumab at a dose of 15 mg/kg once every 3 weeks for 15 or 30 months.	The median PFS: standard duration of bevacizumab: 24.2 months, extended duration of bevacizumab: 26.0 months. No difference was found between patient groups in the median OS.
Gilbert et al. [60]	2023	Phase Ib/II	94 Patients with recurrent epithelial ovarian, fallopian tube, or primary peritoneal cancer, whose most recent platinum-free interval was ≤6 months	Mirvetuximab soravtansine (6 mg/kg adjusted ideal body weight) and bevacizumab (15 mg/kg), intravenously, once every 3 weeks	The median PFS was 8.2 months and the median DOR was 9.7 months

**Table 2:** Clinical trials with bevacizumab in the cervical cancer.

The Name of the Study	The Year of the Study	The Phase of the Study	Research Group	Dose of Bevacizumab	Results
GOG 227C [73]	2009	Phase II trial	46 patients with advanced cervical cancer, (82.6%) 38 of them received prior radiation as well as either one (*n* = 34) or two (*n* = 12) prior cytotoxic regimens for recurrent disease	Bevacizumab: 15 mg/kg every 3 weeks until disease progression or prohibitive toxicity	PFS was 3.4 months and OS—7.3 months
GOG 240 [74]	2014	Phase III trial	452 patients with advanced cervical cancer to chemotherapy with (*n* = 227) or without (*n* = 225) bevacizumab	Bevacizumab: 15 mg/kg	Bevacizumab together with the chemotherapy in patients with metastatic, recurrent or persistent cervical cancer improved the OS, which was 3.7 months higher than in a group without bevacizumab.
GOG 240 [75]	2017	Phase III trial	452 patients with advanced cervical cancer	Bevacizumab administered intravenously at a dose of 15 mg/kg on day 1 in 21-day cycles	Efficacy and tolerability of bevacizumab in the treatment of a advanced cervical cancer.

**Table 3:** Clinical trials with bevacizumab in the endometrial cancer.

Authors of the Study	The Year of the Study	The Phase of the Study	Research Group	Dose of Bevacizumab	Results
Wright et al. [80]	2007	A retrospective analysis	11 patients, including 9 patients with epithelial endometrial carcinomas and 2 with leiomyosarcomas.	Median cumulative dose received by patients was 4.679 mg.	Median PFS was 5.4 months for the entire cohort and 8.7 months for those who achieved clinical benefit, bevacizumab was well tolerated.
Aghajanian et al. [81]	2011	Phase II	56 patients, 29 had received prior radiation.	Treatment consisted of bevacizumab 15 mg/kg intravenously every 3 weeks until disease progression or prohibitive toxicity.	Median PFS and OS were 4.2 and 10.5 months, bevacizumab was well tolerated in recurrent or persistent endometrial cancer.
Alvarez et al. [82]	2012	Phase II	53 patients, 20 had received prior radiation.	Bevacizumab 10 mg/kg every other week.	PFS and OS were 5.6 and 16.9 months, respectively.
Rubinstein et al. [83]	2021	A retrospective analysis	101 patients including 13 grade 1/2 endometrioid, 15 grade 3 endometrioid, 44 serous, 8 carcinosarcoma, and 21 other/mixed histologies.	85 patients started bevacizumab at a dose of 15 mg/kg, 9 started at 10 mg/kg, and 7 started at 7.5 mg/kg, with dosing every 3 weeks.	Median PFS ranged from 2.6 months (2 lines) to 4.9 months (≥4 lines), the median OS was 3.4 years.

## Data Availability

Not applicable.
